# Risk factors and protective factors associated with incident or increase of frailty among community-dwelling older adults: A systematic review of longitudinal studies

**DOI:** 10.1371/journal.pone.0178383

**Published:** 2017-06-15

**Authors:** Zeyun Feng, Marjolein Lugtenberg, Carmen Franse, Xinye Fang, Shanlian Hu, Chunlin Jin, Hein Raat

**Affiliations:** 1 Department of Public Health, Erasmus University Medical Center, Rotterdam, The Netherlands; 2 Shanghai Health Development Research Center (Shanghai Medical Information Center), Shanghai, China; 3 Shanghai Population Development Research Center, Shanghai, China; Nathan S Kline Institute, UNITED STATES

## Abstract

**Introduction:**

Frailty is one of the greatest challenges facing our aging population, as it can lead to adverse outcomes such as institutionalization, hospitalization, and mortality. However, the factors that are associated with frailty are poorly understood. We performed a systematic review of longitudinal studies in order to identify the sociodemographic, physical, biological, lifestyle-related, and psychological risk or protective factors that are associated with frailty among community-dwelling older adults.

**Methods:**

A systematic literature search was conducted in the following databases in order to identify studies that assessed the factors associated with of frailty among community-dwelling older adults: Embase, Medline Ovid, Web of Science, Cochrane, PsychINFO Ovid, CINAHL EBSCOhost, and Google Scholar. Studies were selected if they included a longitudinal design, focused on community-dwelling older adults aged 60 years and older, and used a tool to assess frailty. The methodological quality of each study was assessed using the Quality of Reporting of Observational Longitudinal Research checklist.

**Results:**

Twenty-three studies were included. Significant associations were reported between the following types of factors and frailty: sociodemographic factors (7/7 studies), physical factors (5/6 studies), biological factors (5/7 studies), lifestyle factors (11/13 studies), and psychological factors (7/8 studies). Significant sociodemographic factors included older age, ethnic background, neighborhood, and access to private insurance or Medicare; significant physical factors included obesity and activities of daily living (ADL) functional status; significant biological factors included serum uric acid; significant lifestyle factors included a higher Diet Quality Index International (DQI) score, higher fruit/vegetable consumption and higher tertile of all measures of habitual dietary resveratrol exposure; significant psychological factors included depressive symptoms.

**Conclusions:**

A broad range of sociodemographic, physical, biological, lifestyle, and psychological factors show a longitudinal association with frailty. These factors should be considered when developing interventions aimed at preventing and/or reducing the burden associated with frailty among community-dwelling older adults.

## Introduction

In recent years, frailty has received increasing attention with respect to efforts designed to increase the healthy life expectancy among our aging population and to improve healthcare among the elderly [1]. Although some people remain relatively healthy and resilient with aging, others become more vulnerable to external and/or internal stressors, indicating a state of frailty [2]. Because frailty is associated with negative health outcomes such as institutionalization, hospitalization, and mortality [2, 3], frailty is generally considered a useful concept for clinicians [4].

Currently, no clear consensus exists regarding the definition of frailty [5]. The most widely used definition of frailty was proposed by Fried [6], which states that frailty is “a state of age-related physiological vulnerability resulting from impaired homeostatic reserve and a reduced capacity of the organism to withstand stress”. Moreover, an increasing number of researchers now recognize the multifactorial nature of the concept of frailty [7]. A recently proposed integral conceptual model by Gobbens et al. defines frailty as “a dynamic state affecting an individual who experiences losses in one or more domains of human functioning (physical, psychological, and social), which is caused by the influence of a range of variables and which increases the risk of adverse outcomes” [7]. In our review, we focus both on studies using the physical definition of frailty as well as on studies using the broader definition of frailty that includes psychological and social aspects besides from the physical domain.

Various studies have focused on identifying the factors associated with frailty, particularly sociodemographic factors such as age, gender, and educational level, as well as physical factors such as body weight and activities of daily living (ADL) [8–10]. Recently, however, an increasing number of studies have begun to focus on the role of biological, lifestyle, and psychological factors with respect to frailty, which may provide a more comprehensive view of health disparities among the elderly [11–17]. Identifying the entire range of factors of frailty may be useful for developing interventions designed to prevent and/or reduce the burden that frailty places on the individual and may provide directions for future public health policies [18].

Although reviews of studies regarding frailty have been conducted, these reviews focused primarily on one type (e.g., social, physical, or psychological) of factors [2, 19, 20]. Indeed, only one review attempted to identify a broader range of factors [21]; however, in their review the authors predominantly included cross-sectional studies, thereby making it difficult to examine the putative causal relationship between the factors identified and frailty.

Here, we conducted a systematic review of the results obtained from published longitudinal studies regarding the sociodemographic, physical, biological, lifestyle-related, and psychological risk or protective factors that are associated with incident or increase of frailty in community-dwelling elderly people aged 60 years and above. We focused our analysis on community-dwelling (i.e., independent living) older adults because the early stages of frailty are particularly common within this population; therefore, this population may benefit most from initiatives designed to identify and prevent frailty [7].

## Materials and methods

### Registration

We registered our systematic review protocol at PROSPERO (registration number: RD42016050993; URL: http://www.crd.york.ac.uk/PROSPERO/display_record.asp?ID=CRD42016050993). The PRISMA (Preferred Reporting Items for Systematic Reviews and Meta-Analyses) checklist was used for this review (S1 Table) [22].

### Search strategy

In September 2016, a systematic literature search was conducted in the following databases: Embase, Medline Ovid, Web of Science, Cochrane, PsychINFO Ovid, CINAHL EBSCOhost, and Google Scholar. Several combinations of the following key words were included in the search: “frail”, “community-dwelling”, “biological factors”, “demography”, “sociological factors”, “socioeconomic factors”, “living standard”, and/or “psychological factors”. The search strategy was adapted for each database. The complete search strategies used are presented in S1 File.

### Inclusion criteria

To be included in the first selection round, each study had to meet the following general criteria: *i*) it was an original scientific article; *ii*) the study’s primary objective was to identify at least one sociodemographic, physical, biological, lifestyle, or psychological factor associated with frailty among the elderly; *iii*) it focused on community-dwelling older adults; and *iv*) frailty was the main outcome of the study.

In the second selection round, each study had to meet the following criteria: *i*) it used a longitudinal design, and the association between potential determining factors and frailty was analyzed longitudinally; *ii*) it used a clear definition of frailty; *iii*) it used a certain tool to assess frailty; and *iv*) the study sample was ≥60 years of age at baseline.

### Selection process

In the first selection round, two authors (ZF and XF) independently screened the titles and abstracts based on the general inclusion criteria; they then selected potentially relevant papers. Any discrepancies were resolved by discussion and consensus. The search results were supplemented by reviewing the references cited in key papers.

In the second selection round, the full text of each remaining paper was obtained and independently assessed for eligibility by two authors (ZF and XF) using the inclusion criteria listed above. Any disagreements were discussed with a third author (ML) until consensus was reached.

### Data extraction

Data were extracted from each paper by one author (ZF) using a structured data-extraction form and were recorded in a standardized data-extraction file. The following data were extracted from each paper: first author and year of publication, study design, country, sample size at baseline, sample age at baseline, type of factors included, the frailty assessment tool used, the statistical methodology used, the main results, the sample size at follow-up, and the duration of follow-up. A 100% data check was performed by a second author (ML). Any discrepancies were discussed until consensus was reached.

### Quality assessment

The methodological quality of each study was assessed by one reviewing author (ZF) and then verified by a second reviewing author (CF or XF). Quality was assessed using the Quality of Reporting of Observational Longitudinal Research checklist [23], a 30-item checklist specifically designed for observational longitudinal research. This checklist includes various methodological aspects (e.g., study design, study rationale, population, subject recruitment, measurement and biases, data analysis, and the generalizability of the results) [23]. Each study was assessed for each item; a score of 1 (“YES”) was given if the description was provided, and a score of 0 (“NO”) was given if the description was not clear or not available. In case of a discrepancy between the first and second reviewing authors, a third author (ML or HR) was consulted until consensus was reached. A quality score was then assigned to each study, with a maximum total score of 30 for each study. Following Sing et al. [24], a study was considered to be of ‘adequate quality’ if it received at total score of more than 50% (score>15). Studies receiving scores of >20 were considered as ‘high quality studies’.

### Data synthesis

To assess the factors associated with frailty, all types of factors (sociodemographic, physical, biological, lifestyle, psychological, and other) were assessed and are summarized. We only included factors that were reported in longitudinal multivariable adjusted models; both significant and non-significant associations were evaluated. Studies that reported significant associations but did not provide *p*-values were also included. Data obtained from separate studies using the same cohort data and similar factors were combined and treated as one study.

## Results

### Description of studies

#### Study selection

**Fig 1:**
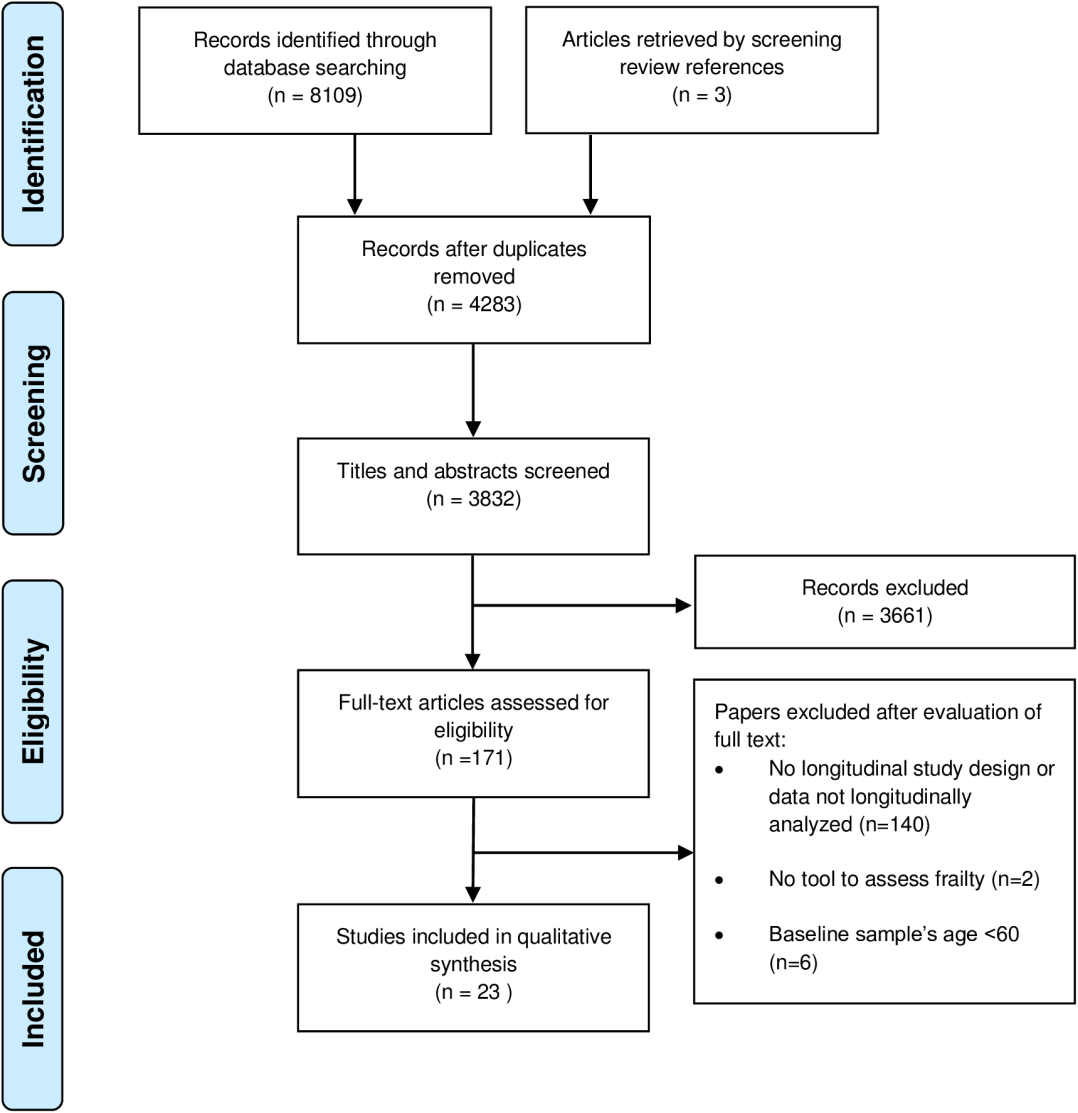
Flow chart of study selection process.

A total of 8109 papers were identified in our initial search of seven databases. After duplicates were removed, 3829 articles remained. The selection process, the number of excluded papers, and the reasons for exclusion are summarized in Fig 1. A total of 23 papers met the inclusion criteria and were included in the analysis.

#### Study characteristics

The year of publication of the included papers ranged from 2005 through 2016. The majority of papers (17 out of 23) were published in 2011 or later; 6 papers were published in 2016. The majority of the included papers originated in a European country (n = 11) or in the United States (n = 9). The number of baseline participants ranged from 624 to 40,657; the majority of studies included >2000 subjects. In 14 studies, the age for selection of participants was over 65 years; in 3 studies, the participants’ age at baseline was over 70 years of age.

The most frequently studied factors were sociodemographic factors (n = 18 studies), followed by lifestyle factors (n = 17 studies), physical factors (n = 13 studies), psychological factors (n = 11 studies), and biological factors (n = 9 studies). Eighteen of the 23 studies used logistic regression to analyze the association between various factors and frailty. The most frequently used criteria for assessing frailty were Fried’s criteria for frailty (n = 15 studies) or a modified version of Fried’s criteria for frailty (n = 5 studies), the FRAIL scale (n = 2 studies), and the Rockwood frailty index (n = 1 studies). The follow-up period ranged from 3 years to 13 years. The general characteristics of the included studies are summarized in Table 1.

**Table 1:** Characteristics of the included studies (n = 23).

First author and citation	Publication Year	Country	Sample size at baseline	Sample age at baseline, years	Type(s) of factor(s) included in the study*	Frailty assessment tool	Statistical technique(s)	Sample size at follow-up	Years of follow-up
Woods, N.F. [25]℄	2005	United States	40,657	65–79	• Sociodemographic factors • Physical factors • Lifestyle factors • Psychological factors • Other factors	Fried’s frailty criteria	- Bivariate analysis and multivariate logistic regression	28,181	5.9 (Average)
Semba, R.D. [26]	2006	United States	766 (women)	≥65	• Sociodemographic factors • Physical factors • Biological factors • Lifestyle factors	Fried’s frailty criteria	- Bivariate analysis	1002	3
Cawthon, P.M. [27]	2009	United States	1469	≥65	• Sociodemographic factors • Physical factors • Biological factors • Psychological factors	Fried’s frailty criteria (CHS)	- Ordinal logistic regression	1245	4.1
Gruenewald, T.L. [28]	2009	United States	1189	70–79	• Sociodemographic factors • Physical factors • Biological factors	Fried’s frailty criteria	- Multivariable ordinal logistic regression	1103	3
Ottenbacher, K.J. [29]	2009	United States	2049	≥65	• Sociodemographic factors • Physical factors • Lifestyle factors • Psychological factors	Fried’s frailty criteria	- Multiple linear regression	2049	10
Hyde, Z. [30]	2010	Australia	3616 (men)	70–88	• Sociodemographic factors • Biological factors • Lifestyle factors	FRAIL scale	- Binary logistic regression models	1586	7
Aranda, M.P. [8]	2011	United States	2069	≥75	• Sociodemographic factors • Physical factors • Lifestyle factors • Psychological factors	Modified version of Fried’s frailty criteria	- Cumulative logistic regression model	1447	2
Ensrud K.E. [31]	2011	United States	1606 (men)	65	• Sociodemographic factors • Physical factors • Lifestyle factors • Biological factors	Fried’s frailty criteria	- Partial proportional odds models	1128	4.6
Lakey S.L. [32]℄	2012	United States	33,324	65–79	• Psychological factors	Fried’s frailty criteria	- Multivariate logistic regression	27,652	3
Talegawkar, S.A. [33]	2012	Italy	1155	≥65	• Sociodemographic factors • Physical factors • Lifestyle factors	Fried’s frailty criteria	- First-order transition models	690	6
Baylis, D. [12]	2013	United Kingdom	717	65–70	• Biological factors	Fried’s frailty criteria	- Pearson’s correlation coefficients Cox’s proportional hazards models Logistic regression models Bonferroni correction	254	10
Hoogendijk, E.O. [9]	2014	The Netherlands	1205	≥65	• Sociodemographic factors • Physical factors • Biological factors • Lifestyle factors • Psychological factors	Fried’s frailty criteria	- Longitudinal logistic regression analyses	N = 1205 at T1; N = 909 at T2; N = 659 at T3; N = 433 at T4; N = 309 at T5	Every 3 years
León-Muñoz, L.M. [34]	2014	Spain	2519	≥60	• Sociodemographic factors • Physical factors • Lifestyle factors • Psychological factors	Fried’s frailty criteria	- Logistic regression	1815	3.5 (Average)
Myers, V. [10]	2014	Israel	1626	≤65	• Sociodemographic factors • Physical factors • Lifestyle factors • Psychological factors • Other factor	The Rockwood frailty index	- Multivariable logistic regression Models, analysis of variance (ANOVA)	1151	10–13
Chan, R. [35]	2015	China, Hong Kong	4000	≥65	• Lifestyle factors	FRAIL scale	- Logistic regression models	2724	3.9 (Average)
Lana, A. [36]	2015	Spain	2614	≥60	• Lifestyle factors	Modified version of Fried’s frailty criteria	- Logistic regression	1871	3 (Average)
Rabassa, M. [37]	2015	Italy	1260	≥65	• Sociodemographic factors • Physical factors • Biological factors • Lifestyle factors • Psychological factors	Fried’s frailty criteria	- Multinomial logistic regression	529 (3-year follow-up), 442 (6-year follow-up), 322 (9-year follow-up)	3, 6, and 9 years of follow-up
García-Esquinas, E. [14]	2016	Spain	Cohort 1 = 2614 Cohort 2 = 1214 Cohort 3 = 695	≥60	• Sociodemographic factors • Lifestyle factors	Modified version of Fried’s frailty criteria	- Logistic regression, Chi-square- based Q statistic	Cohort 1 = 1872 Cohort 2 = 581 Cohort 3 = 473	2.5
García-Esquinas, E. [38]	2016	Spain	2614	≥60	• Sociodemographic factors • Biological factors • Lifestyle factors	Fried’s frailty criteria	- Logistic regression	2198	3.5 (Avergae)
McHugh, J.E. [15]	2016	Ireland	624	≥60 (72.75 mean)	• Sociodemographic factors • Physical factors • Psychological factors	Modified version of Fried’s frailty criteria	- Logistic regression	447	2
Monin, J. [13]	2016	United States	5201	≥65	• Psychological factors	Modified version of Fried’s frailty criteria	- ANOVA	5888	7
Ortolá, R. [16]^※^	2016	Spain	2086	≥60	• Sociodemographic factors • Lifestyle factors	Fried’s frailty criteria	- Logistic regression	2404	3.3 (Avergae)
Sandoval-Insausti, H. [17]^※^	2016	Spain	2614	≥60	• Sociodemographic factors • Lifestyle factors	Fried’s frailty criteria	- Logistic regression	1822	3.5 (Avergae)

CHS, Cardiovascular Health Study Index (Fried’s frailty criteria); FRAIL, Fatigue, Resistance, Ambulation, Illness and Loss of Weight Index; NA, not available.

*: Please note: Not all factors were used in longitudinal analysis, only factors that were included in the longitudinal analyses were eligible for the data extraction for Table 2

℄: The Woods et al. (2005) and Lakey et al. (2012) studies are both based on the same WHI-OS cohort study

※: Ortolá, R. & Sandoval-Insausti, H. used the same database (Seniors-ENRICA cohort)

### Methodological quality of the included studies

The total quality scores of the studies ranged from 13 to 24 (S2 Table). Twenty-two of the 23 included studies scored >15 and were therefore considered to be of adequate quality. Five of the 23 included studies scored >20 were considered to be high quality studies.

Each of the included studies described the target population, study population, dates when conducted, eligibility criteria, number of participants at baseline, number of participants at follow-up, and type of analyses. The majority of the studies described the study setting (22/23), the objective of the study (21/23) and/or the sample frame (21/23). Only one study did not report relative effect sizes. None of the 23 studies reported their justification for the number of participants and none of the studies stated their reasons for refusal to consent. Only one of the 23 studies compared consenters with non-consenters. The results of our quality assessment analysis are presented in S2 Table.

### Factors associated with frailty

#### Sociodemographic factors

Seven of the 23 studies assessed sociodemographic factors. All of these seven studies reported at least one significant association between sociodemographic factors and frailty. The most frequently studied sociodemographic variables were age, gender, and education (Table 2).

**Table 2:** Associations (from fully adjusted models) between various types of factors and frailty.

		Author (year of publication)	Significant association (*p*<0.05)	Type of association (positive or negative)
**Sociodemographic factors**				
Age (older)		Aranda [8] (2011), Myers [10] (2014), Woods [25] (2005), McHugh [15] (2016), Ottenbacher [29] (2009), Semba [26] (2006)♀	Yes	Positive
Gender (female)		Myers [10] (2014), Ottenbache[29](2009)	Yes	Positive
		Aranda [8] (2011), McHugh [15] (2016)	No	N/A
Education level (lower)		Woods [25] (2005)	Yes	Positive
		Aranda [8] (2011), Myers [10] (2014), Hoogendijk [9] (2014)	No	N/A
Income:				
	*Low income*	Myers [10] (2014), Woods [25] (2005)	Yes	Positive
	*Financial strain*	Aranda [8] (2011)	No	N/A
	*Medium income (compared to low income)*	Hoogendijk [9] (2014)	No	N/A
	*High income (compared to low income)*	Hoogendijk [9] (2014)	Yes	Negative
Ethnic background (African-American)		Woods [25] (2005)	Yes	Positive
Neighborhood:				
	*High-density*	Aranda [8] (2011)	Yes	Positive
	*SES (lower or middle tertile)*	Myers [10] (2014)	Yes	Positive
Partner status (married or having a partner)		Ottenbacher [29] (2009), Hoogendijk [9] (2014)	No	N/A
Living alone		Woods [25] (2005)	Yes	Negative
		Aranda [8] (2011)	No	N/A
Private insurance or Medicare		Aranda [8] (2011)	Yes	Positive
Network size		Hoogendijk [9] (2014)	No	N/A
Pre-MI employment (full-time or part-time vs. none)		Myers [10] (2014)	No	N/A
**Physical factors**				
Weight:				
	*BMI (BMI <18*.*5 or BMI 25*.*0–29*.*9 or BMI ≥30)(compared to BMI 18*.*5–24*.*9)*	Woods [25] (2005)	Yes	Positive
	*BMI (continuous)*	Ottenbacher [29] (2009)	Yes	Positive
	*Underweight (BMI <18*.*5)*	Aranda [8] (2011)	No	N/A
	*Obese (BMI ≥30*.*0)*	Myers [10] (2014), Woods [25] (2005), Hoogendijk [9] (2014)	Yes	Positive
ADL functional status		Aranda [8] (2011), Ottenbacher [29] (2009)	Yes	Positive
Reduced function of extremities		Ottenbacher [29] (2009)	Yes	Positive
Higher allostatic load (AL) (dysregulation across multiple physiological systems)		Gruenewald [28] (2009)	Yes	Positive
Q-wave myocardial infarction		Myers [10] (2014)	No	N/A
Early revascularization		Myers [10] (2014)	No	N/A
Hypertension		Myers [10] (2014)	No	N/A
**Biological factors**				
Immune-endocrine biomarkers:				
	*Higher white cell count*, *higher numbers of monocytes or lymphocytes*, *higher albumin level*, *lower level of DHEASa*, *higher cortisol*:*DHEAS ratio*	Baylis [12] (2013)	Yes	Positive
	*ESR*, *neutrophils*, *hemoglobin*, *TSH*, *T4*, *IL-1β*, *IL-6*, *IL-10*, *cortisol*	Baylis [12] (2013)	No	N/A
	*Lower free testosterone*	Hyde [30] (2010) ♂	Yes	Positive
	*Level of testosterone* ♂	Baylis [12] (2013), Cawthon [27] (2009)	No	N/A
	*CRP highest tertile*	Baylis [12] (2013)	No	N/A
		Hoogendijk [9] (2014)	Yes	Positive
	*Lower level of 25(OH)D*	Ensrud [31] (2011)	No	N/A
		Hoogendijk [9] (2014)	Yes	Positive
	*SHBG concentration*	Baylis [12] (2013), Cawthon [27] (2009), Hyde [30] (2010) ♂	No	N/A
	*Level of estradiol*, *bioavailable estradiol*	Cawthon [27] (2009)	No	N/A
	*Lowest tertile of IGF-1*	Hoogendijk [9] (2014)	No	N/A
Women in the lowest quartile of serum carotenoids		Semba [26] (2006) ♀	Yes	Positive
Various micronutrient deficiencies (compared with no deficiencies)		Semba [26] (2006) ♀	Yes	Positive
Serum uric acid		García-Esquinas [14] (2016) ♂	Yes	Positive
**Lifestyle factors (health-related factors)**				
Dietary patterns:				
Mediterranean:				
	*High adherence to Mediterranean-style diet*	Talegawkar [33] (2012)	Yes	Negative
	*Higher MDS*	León-Muñoz [34] (2014)	Yes	Negative
		Chan [35] (2015)	No	N/A
	*Higher MEDAS*	León-Muñoz [34] (2014)	No	N/A
Other dietary patterns:				
	*Higher DQI score*	Chan [35] (2015)	Yes	Negative
	*“Vegetables-fruits” pattern or “meat-fish” pattern*	Chan [35] (2015)	No	N/A
	*Higher fruit/vegetable consumption (three portions of fruit/day and two portions of vegetables/day)*	García-Esquinas [38] (2016)	Yes	Negative
Individual (dietary/lifestyle) factors:				
Smoking		Woods [25] (2005), Ottenbacher [29] (2009), Hoogendijk [9] (2014)	Yes	Positive
		Myers [10] (2014),Semba [26] (2006) ♀	No	N/A
Alcohol intake:				
	*Drinking alcohol only with meals/MDP*	Ortolá [16] (2016)	Yes	Negative
	*Moderate alcohol intake*	Woods [25] (2005)	Yes	Negative
	*Heavy drinker (compared to non-drinker)*	Ortolá [16] (2016)	Yes	Negative
	*Alcohol use (number of alcohol consumptions a week*, *0–77)*	Hoogendijk [9] (2014)	No	N/A
Protein				
	*Intake of total protein*, *animal protein*, *MUFAs (higher)*	Sandoval-Insausti [17] (2016)	Yes	Negative
	*Intake of vegetable protein*, *SFAs*, *ALA*, *LA*, *carbohydrates*, *simple sugars*, *polysaccharides*, *long-chain ω-3 fatty acids*	Sandoval-Insausti [17] (2016)	No	N/A
Milk & yogurt intake				
	*Low-fat milk and yogurt*	Lana [36] (2015)	Yes	Negative
	*Whole milk*, *whole-fat yogurt*, *low-fat yogurt*, *cheese*, *whole milk OR yoghurt*	Lana [36] (2015)	No	N/A
Higher tertile of habitual dietary resveratrol exposure (TDR, TUR, and TDR+TUR)		Rabassa [37] (2015)	Yes	Negative
**Psychological factors**				
Depressive symptoms:‡				
	*Higher levels of depressive symptoms*	[Woods [25] (2005) & Lakey [32] (2012)], Hoogendijk [9] (2014)	Yes	Positive
	*Spouse’s depression*	Monin [13] (2016)	Yes	Positive
Higher score of positive affect subscale of the CES-D		Ottenbacher [29] (2009)	Yes	Positive
Lower MMSE score /impaired cognitive function		Ottenbacher [29] (2009) Aranda [8] (2011), Hoogendijk [9] (2014)	Yes	Positive
Self-rated health:				
	*Poor self-rated health*	Myers [10] (2014)	Yes	Positive
	*Average self-rated health*	Myers [10] (2014)	No	N/A
Negative affect		Ottenbacher [29] (2009)	Yes	Positive
Mastery (5–25)		Hoogendijk [9] (2014)	Yes	Negative
Emotional support		Aranda [8] (2011), Hoogendijk [9] (2014)	No	N/A
Self-efficacy (12–60)		Hoogendijk [9] (2014)	No	N/A
Anxiety		McHugh [15] (2016)	No	N/A
Neuroticism		McHugh [15] (2016)	No	N/A
**Other factors**				
Number of falls in the previous 12 months (≥1)		Woods [25] (2005)	Yes	Positive
Hormone use		Woods [25] (2005)	Yes	Positive
Medication use (ACE inhibitor, aspirin, beta-blocker)		Myers [10] (2014)	No	N/A

^♀^: sample included only women

^♂^: sample included only men or was based on data from men only

^‡^: The Woods et al. (2005) and Lakey et al. (2012) studies are both based on the same WHI-OS cohort study and are treated as one study when reporting the same variable

N/A: not applicable. ACE: angiotensin-converting enzyme; ADL: activities of daily living; ALA: α-linolenic acid; AL: allostatic load; BMI: body mass index; CES-D: Center for Epidemiologic Studies Depression; CRP: C-reactive protein; DHEAS: dehydroepiandrosterone sulfate; DQI: Diet Quality Index-International; ESR: erythrocyte sedimentation rate; IGF-1:insulin-like growth factor 1; IL-1β: human interleukin-1β; IL-6: human interleukin-6; IL-10: human interleukin-10; LA: linoleic acid; MDS: Mediterranean Diet Score; MEDAS: Mediterranean Diet Adherence Screener; MDP: Mediterranean drinking pattern; MI: myocardial infarction; MUFAs: monounsaturated fatty acids; MMSE: Mini-Mental State Exam; N/A: not applicable; SES: socioeconomic status; SHBG: sex hormone-binding globulin; SFAs: saturated fatty acids; TDR: total dietary resveratrol; TSH: thyroid-stimulating hormone; TUR: total urinary resveratrol; T4: free thyroxine; 25(OH)D: 25-hydroxyvitamin D.

Older age (p<0.05, p<0.05, p<0.001, p<0.001, p<0.05, p<0.001), female gender (p<0.05, p<0.05), lower education level (p<0.01, p<0.05), lower income (p<0.05, p<0.001), African-American ethnic background (p<0.01), living in a high-density neighborhood (p<0.05), socioeconomic status of the neighborhood (lower or middle tertile) (p<0.05), and private insurance or Medicare (p<0.05) were all positively significantly associated with frailty (Table 2). However, two studies reported no significant association between gender and frailty, three studies reported no significant association between education level and frailty, and one study reported no significant association between income and frailty.

One study reported a significant negative association between higher income and frailty (p<0.05), one study reported a significant negative association between living alone (p<0.001) and frailty; however, another study reported no significant association between living alone and frailty. Lastly, no significant associations were reported between frailty and medium income, partner status, network size, or employment.

#### Physical factors

Six of the 23 studies assessed physical factors. Five of these six studies reported at least one significant association between physical factors and frailty (Table 2). The most frequently studied physical factor was weight.

Body mass index (BMI) (p<0.001), obesity (p<0.05, p<0.001, p<0.05), activities of daily living (ADL) functional status (p<0.05, p<0.05), reduced functions of extremities (p<0.05), and higher allostatic load (AL) (p<0.05) were all significantly positively associated with frailty. However, one study reported no significant association between underweight (BMI <18.5) and frailty. Lastly, no significant associations were reported between frailty and Q-wave myocardial infarction (MI), early revascularization, or hypertension.

#### Biological factors

Seven of the 23 studies assessed biological factors, and five of these seven studies reported at least one significant association between a biological factor and frailty (Table 2). The most frequently studied biological factor was sex hormone-binding globulin (SHBG) concentration.

Positive associations were reported between the following immune-endocrine markers and frailty: higher white cell count, higher number of monocytes, higher number of lymphocytes, higher albumin level, lower levels of dehydroepiandrosteron e sulfate (DHEAS), cortisol:DHEAS ratio (p<0.05), lower free testosterone (p<0.05), C-reactive protein (CRP) highest tertile (p<0.05), and lower level of circulating 25-hydroxyvitamin D (25(OH)D) (p<0.05). However, one study found no significant association between testosterone level and frailty, one study found no significant association between CRP highest tertile and frailty and one study found no significant association between lower level of 25(OH)D and frailty.

No significant association were reported between the following immune-endocrine biomarkers and frailty: erythrocyte sedimentation rate, neutrophils, hemoglobin, thyroid-stimulating hormone, free thyroxine, interleukin-1β, interleukin-6, interleukin-10, cortisol, SHBG concentration, level of estradiol levels, or bioavailable estradiol, and lowest tertile of insulin-like growth factor 1 (IGF-1).

Besides from immune-endocrine biomarkers significant positive associations were also reported between frailty and women in the lowest quartile of serum carotenoids (p<0.05), various micronutrient deficiencies (p<0.05) and serum uric acid (p<0.01).

#### Lifestyle factors (health-related factors)

Thirteen of the 23 studies measured lifestyle factors. Eleven of these 13 studies found at least one significant association between specific lifestyle factors and frailty (Table 2). The most frequently studied lifestyle factors were dietary patterns, smoking and alcohol consumption.

With respect to a Mediterranean dietary pattern significant negative associations were found between frailty and high adherence to a Mediterranean-style diet (p<0.05) and a higher Mediterranean Diet Score (MDS) (p<0.05). However, another study reported no significant association between MDS and frailty. In addition, no significant association was found between frailty and a higher Mediterranean Diet Adherence Screener (MEDAS) score.

For other dietary patterns, a significant negative association was found between frailty and a higher Diet Quality Index International (DQI) score (p<0.05). No significant association was reported between frailty and a “vegetables-fruits” pattern or “meat-fish” pattern.

With respect to the individual factors, positive associations were reported between frailty and smoking in three studies (p<0.001, p<0.05, p<0.05). However, two other studies found no significant association between smoking and frailty. For alcohol intake significant negative associations were found between frailty and drinking alcohol only with meals, moderate alcohol consumption, and being a heavy drinker (compared with non-drinkers) (p<0.05, p<0.01, p<0.001). One study, however, reported no significant association between alcohol intake and frailty.

Significant negative associations were found between frailty and higher consumption of fruit/vegetable (p<0.01) and protein consumption (including total proteins, animal proteins, and higher MUFAs) (p<0.01). Lastly, no significant associations were reported between frailty and the consumption of vegetable-based protein, saturated fatty acids (SFAs), α-linolenic acid (ALA), linoleic acid (LA), carbohydrates, simple sugars, polysaccharides, or long-chain ω-3 fatty acids.

With respect to milk and yoghurt intake one study found that the consumption of low-fat milk and yogurt was negatively associated with frailty (p<0.05). However, another study reported that frailty was not significantly associated with the consumption of whole milk, whole-fat yogurt, low-fat yogurt, cheese or whole milk OR yoghurt.

Finally, one study reported that a higher tertile of habitual dietary resveratrol exposure (TDR, TUR, and TDR+TUR) was negatively associated with frailty (p<0.05).

#### Psychological factors

Eight of the 23 studies measured psychological factors; the most frequently studied psychological factor was depressive symptoms. Seven of these eight studies reported at least one significant association between specific psychological factors and frailty (Table 2).

Positive associations were found between frailty and a higher level of depression (p<0.001, p<0.05), the spouse’s depression (p<0.01), a higher positive affect subscale score on the CES-D (Center for Epidemiologic Studies Depression) (p<0.05), lower Mini-Mental State Exam (MMSE) score/impaired cognitive function (p<0.05, p<0.05, p<0.05), poor self-rated health (p<0.05), and negative affect (p<0.05). In contrast, one study found no significant association between average self-rated health and frailty.

A significant negative association was found between mastery and frailty (p<0.05). Finally, no significant associations were found between frailty and emotional support, self-efficacy, anxiety or neuroticism.

#### Other factors

Two of the 23 studies measured other factors. Positive associations were found between frailty and number of falls (≥1) in the previous 12 months (p<0.001), and hormone use (p<0.001). No significant association was found between frailty and medication use.

## Discussion

The aim of this systematic review was to provide an overview of published results from longitudinal studies regarding the risk factors and protective factors associated with incident or increase of frailty by taking into account the entire spectrum of putative factors. We found that a wide variety of sociodemographic, physical, biological, lifestyle, and psychological factors are associated with frailty.

Consistent with a previous review regarding factors associated with frailty [21], our findings indicate that sociodemographic factors (e.g., older age, ethnical background), physical factors (e.g., obesity, ADL), and psychological factors (depressive symptoms) contribute to frailty. However, we also found that other, less commonly studied sociodemographic factors including neighborhood and access to private insurance, biological and lifestyle factors including serum uric acid, various micronutrient deficiencies, lifestyle factors including a higher Diet Quality Index International (DQI) score and higher tertile of all measures of habitual dietary resveratrol exposure are significantly associated with frailty. Each of these factors may play a specific role in the development of frailty or in a particular domain of frailty [2]. Although the majority of older studies focused primarily on the sociodemographic factors of frailty, the more recently published studies included in our review tended to focus more on lifestyle-related, biological, and psychological factors associated with frailty. This trend may reflect an increasing attention towards modifiable risk factors for frailty which can be treated or changed through behavioral interventions.

However, in our review, for a number of factors we found a significant association with frailty in certain studies and no significant association in other studies. There may be several explanations for this. Firstly, each study has a distinct study population with distinct characteristics that may explain why an association is present or not. Secondly, the absence of an association may be due to a lack of power in the study. Thirdly, the number of years of follow-up and the specific frailty assessment tool that was applied, may explain why there are differences regarding the results between studies. More research in varied large populations, using the same design and measurement tools, is needed to confirm the presence or absence of associations where we have varying results so far.

Our results indicate that several biological factors play a role in frailty. One such factor is serum uric acid which may be due to several possible mechanisms [14]. First, empirical evidence suggests that uric acid is both a potent pro-inflammatory factor and a pro-oxidant in the human body [39], and such factors have been reported in frail older adults [40, 41]. Secondly, the concentration of uric acid has been linked to endothelial dysfunction, which can increase the risk of becoming frail [42]. With respect to testosterone, the results of our review were not consistent: we found a positive association between (lower free) testosterone and frailty in one study but found no significant association between level of testosterone in two other studies. Although the biological mechanisms underlying the association between testosterone and frailty are not well understood, testosterone supplementation has been proposed as a possible treatment for frailty, given that testosterone can increase muscle mass and strength by increasing protein synthesis [43]. More studies are needed to determine the exact association between and underlying mechanisms of testosterone and frailty.

A strength of our study is the unique design in which we included only longitudinal studies with relatively large sample sizes and long follow-up periods. While causality cannot be claimed from observational studies, measurement of factors several years prior to assessment of frailty does indicate the direction of the relationship, suggesting that these factors may affect future frailty status. Another strength of this review is that we also examined the non-significant associations reported in the included studies, thereby minimizing the effect of reporting bias [44]. Moreover, the overall quality of the studies included in our review was quite high; therefore, excluding the relatively few low-quality studies from our analysis would not likely change our main results. Nevertheless, our results highlight the need for further high-quality longitudinal studies that focus on the association between frailty and physical, biological, lifestyle, and psychological factors. Specific risk factors within these groups deserve further attention as well, while some risk factors were more often studied such as weight, smoking and diet other specific risk factors such as physical exercise were not studied.

A possible limitation of our review is that the vast majority (19 out of 23) of the studies utilized Fried’s frailty criteria or a modified version to assess frailty [8, 9, 12–17, 25–29, 31–34, 37, 38], even though we did not specifically limit our inclusion criteria to a certain assessment tool. This likely reflects the fact that Fried’s frailty tool is the most traditional and most widely used tool for assessing frailty. However, because Fried’s frailty criteria only focus on the physical aspects of frailty, relevant psychological and social components of frailty may not have been considered in the studies that used this tool [7]. However, some of the factors assessed (e.g., specific psychological components such as a decline in mood) were labeled as risk factors, rather than components, of frailty in the studies included in our review. This possible overlap should therefore be taken into consideration when including studies that also assess the social and psychological components of frailty [2].

Taken together, our results suggest that several directions for future research should be pursued. First, future studies could include social and psychological components of frailty in addition to physical factors, thereby facilitating discovery of the effects of various types of factors on various frailty domains. Moreover, future reviews could focus on examining how these factors might be used to predict frailty in the short, medium, and long term [2]. We also recommend that future studies investigate the association between relevant factors and frailty, using assessment tools which include not only physical, psychological, and social components to assess frailty in community-dwelling older adults [5]. Several studies have already demonstrated the high validity and reliability of using such a tool within this population [5, 45, 46].

## Conclusions

Determining the factors that have a longitudinal association with frailty is essential for developing interventions designed to prevent and/or reduce the healthcare burdens experienced by frail elderly people. This review demonstrates that several types of risk and protective factors are longitudinally associated with incident or increase of frailty among community-dwelling older adults. In addition to sociodemographic factors, several biological factors, lifestyle-related factors, and psychological factors appear to play a significant role in the development of frailty. These factors should therefore be taken into consideration when developing programs to prevent frailty in community-dwelling older adults.

## Supporting information

S1 Table PRISMA checklist. (PDF)

S2 Table Quality assessment results of the included studies. (PDF)

S1 File The complete search strategies for all databases. (PDF)
