# Supplementary material for: Risk factors and protective factors associated with incident or increase of frailty among community-dwelling older adults: A systematic review of longitudinal studies
Source: PLoS One. 2017 Jun 15;12(6):e0178383. doi: 10.1371/journal.pone.0178383 (PMC5472269; doi:10.1371/journal.pone.0178383)
Supplement: S2 Table — (PDF) [file pone.0178383.s002.pdf]

S2 Table. Quality assessment results of the included studies

NO.	1	2	3	4	5	6	7	8	9	10	11	12	13	14	15	16	17	18	19	20	21	22	23
1. Objectives stated	Y	Y	Y	Y	Y	Y	Y	Y	Y	Y	Y	Y	N	N	Y	Y	Y	Y	Y	Y	Y	Y	Y
2. Target population defined	Y	Y	Y	Y	Y	Y	Y	Y	Y	Y	Y	Y	Y	Y	Y	Y	Y	Y	Y	Y	Y	Y	Y
3. Sampling frame defined	Y	Y	Y	Y	Y	Y	Y	Y	Y	Y	Y	Y	Y	N	Y	Y	Y	Y	Y	Y	Y	Y	N
4. Study population defined?	Y	Y	Y	Y	Y	Y	Y	Y	Y	Y	Y	Y	Y	Y	Y	Y	Y	Y	Y	Y	Y	Y	Y
5. Study setting mentioned	Y	Y	Y	Y	Y	Y	Y	Y	Y	Y	Y	Y	Y	Y	Y	Y	Y	Y	Y	N	Y	Y	Y
6. Dates when conducted stated	Y	Y	Y	Y	Y	Y	Y	Y	Y	Y	Y	Y	Y	Y	Y	Y	Y	Y	Y	Y	Y	Y	Y
7. Eligibility criteria stated	Y	Y	Y	Y	Y	Y	Y	Y	Y	Y	Y	Y	Y	Y	Y	Y	Y	Y	Y	Y	Y	Y	Y
8. Number of participants justified	N	N	N	N	N	N	N	N	N	N	N	N	N	N	N	N	N	N	N	N	N	N	N
9. No. meeting eligibility criteria stated	Y	Y	Y	N	N	N	N	N	N	Y	N	Y	N	N	Y	Y	N	N	Y	Y	Y	Y	N
10. Reasons for eligible stated	N	N	N	N	N	Y	N	N	N	N	N	Y	N	N	Y	Y	N	N	Y	Y	Y	Y	Y
11. No. of consenters stated	N	N	Y	Y	Y	Y	N	Y	Y	Y	N	Y	N	N	N	Y	Y	N	Y	Y	Y	Y	N
12. Reasons for refusal to consent stated	N	N	N	N	N	N	N	N	N	N	N	N	N	N	N	N	N	N	N	N	N	N	N
13. Consenters compared with no consenters	N	N	N	N	N	N	N	N	N	N	N	N	N	N	N	Y	N	N	N	N	N	N	N
14. No. of participants at beginning stated?	Y	Y	Y	Y	Y	Y	Y	Y	Y	Y	Y	Y	Y	Y	Y	Y	Y	Y	Y	Y	Y	Y	Y
15. Methods of data collection stated?	Y	Y	Y	Y	Y	Y	N	Y	Y	Y	N	Y	Y	Y	Y	Y	Y	Y	Y	Y	Y	Y	Y
16. Validity of measurement methods mentioned	N	N	N	N	N	N	Y	Y	Y	N	N	N	N	N	N	N	N	N	Y	Y	Y	Y	Y
17. Confounders mentioned	N	Y	N	Y	Y	N	N	Y	Y	Y	N	Y	Y	Y	Y	N	Y	Y	Y	Y	N	Y	N
18. No. of participants at each stage/wave specified	Y	Y	Y	Y	Y	Y	Y	Y	Y	Y	Y	Y	Y	Y	Y	Y	Y	Y	Y	Y	Y	Y	Y
19. Reasons for loss to follow-up	N	Y	N	Y	N	Y	N	N	N	N	N	Y	Y	Y	N	Y	Y	N	Y	Y	N	Y	Y
20. Missingness of data items mentioned?	N	N	N	N	Y	Y	Y	Y	N	Y	Y	Y	Y	Y	Y	N	N	Y	Y	Y	N	Y	Y
21. Type of analyses stated?	Y	Y	Y	Y	Y	Y	Y	Y	Y	Y	Y	Y	Y	Y	Y	Y	Y	Y	Y	Y	Y	Y	Y
22. “Longitudinal” analysis methods stated	Y	Y	Y	Y	N	Y	Y	N	N	N	Y	N	N	N	N	Y	N	N	Y	Y	Y	Y	Y
23. Absolute effect sizes reported	Y	Y	Y	Y	Y	Y	N	Y	Y	Y	N	N	N	Y	Y	Y	Y	N	Y	Y	Y	Y	Y
24. Relative effect sizes reported	Y	Y	Y	Y	Y	Y	Y	Y	Y	Y	N	Y	Y	Y	Y	Y	Y	Y	Y	Y	Y	Y	Y
25. Loss to follow-up taken for analysis	N	N	N	Y	N	Y	N	N	N	N	N	N	N	N	Y	N	Y	N	N	N	N	Y	N
26. Confounders accounted for in analyses	N	Y	Y	Y	Y	N	N	Y	Y	Y	N	N	Y	Y	Y	Y	Y	Y	Y	Y	Y	Y	N
27. Missing data accounted for in the analyses	N	N	N	N	N	N	Y	N	N	N	Y	N	N	N	Y	N	N	N	N	N	N	N	N
28. Impact of biases assessed	Y	N	N	Y	Y	N	N	Y	N	Y	N	N	Y	Y	N	Y	Y	Y	Y	Y	Y	Y	Y
29. Results related back to the target population	N	N	Y	N	Y	N	Y	N	Y	N	N	Y	Y	N	Y	Y	Y	Y	N	Y	N	N	Y
30. Discussion of generalizability	N	N	Y	N	N	N	Y	N	Y	N	N	N	Y	N	Y	Y	N	N	N	Y	N	N	Y
Total Score	16	18	19	20	19	19	17	19	19	19	13	19	18	16	22	23	20	17	23	24	20	24	20

1.Aranda (2011), 2. Ensrud (2011), 3. Talegawkar (2012), 4. Baylis (2013), 5. León-Muñoz (2014), 6. Hoogendijk (2014), 7. Myers (2014), 8. Lana (2015), 9. Chan (2015), 10. Garcia-Esquinas[2] (2016), 11. Monin (2016), 12. Garcia-Esquinas[1] (2016), 13. Ortola (2016), 14. Sandoval-Insausti (2016), 15. Woods (2005), 16. Semba (2006), 17. Ottenbacher (2009), 18. Lakey (2012), 19. Cawthon (2009), 20. Gruenewald (2009), 21. Hyde (2010), 22. Rabassa (2015), 23. McHugh (2016).
