# Supplementary material for: Risk factors and protective factors associated with incident or increase of frailty among community-dwelling older adults: A systematic review of longitudinal studies
Source: PLoS One. 2017 Jun 15;12(6):e0178383. doi: 10.1371/journal.pone.0178383 (PMC5472269; doi:10.1371/journal.pone.0178383)
Supplement: S1 File — (PDF) [file pone.0178383.s003.pdf]

S1 File. The complete search strategies for all databases

**Databases**	**Hits**	**After duplication**
Embase.com	2400	2346
Medline Ovid	2095	532
Web of science	1787	484
Cochrane	230	17
PsycINFO Ovid	496	100
CINAHL EBSCOhost	901	261
Google scholar	200	89
**Total**	**8109**	**3829**

Embase.com 2400

('independent living'/exp OR 'community'/de OR 'community living'/de OR 'home'/de OR (((independent* OR at-home) NEAR/3 living) OR (aging NEAR/3 place) OR (communit* NEAR/6 (dwell* OR living OR resid*)) OR 'liv* alone'):ab,ti) AND ('frail elderly'/de OR (frail*):ab,ti) AND ('biological factor'/de OR 'social aspect'/de OR 'social status'/exp OR 'social behavior'/exp OR 'psychological aspect'/de OR demography/de OR age/de OR gender/de OR 'sex difference'/de OR 'social determinants of health'/de OR 'income'/de OR 'lowest income group'/de OR 'socioeconomics'/de OR 'educational status'/de OR 'body equilibrium'/de OR depression/de OR 'anxiety'/de OR 'balance disorder'/exp OR 'cognitive defect'/exp OR 'cognition'/exp OR 'daily life activity'/exp OR 'physical activity'/de OR disability/de OR 'disabled person'/de OR 'physical mobility'/de OR 'physical performance'/de OR 'walking'/de OR 'comorbidity'/de OR risk/exp OR 'self concept'/de OR 'self report'/de OR questionnaire/de OR 'assessment of humans'/exp OR 'motor dysfunction'/de OR 'walking difficulty'/de OR (((biological* OR physical* OR social* OR psycholog* OR functional* OR clinical* OR living OR residence* OR age OR gender OR sex OR psychiat* OR neuropsychiat* OR sociological*) NEAR/10 (factor* OR aspect* OR characteristic* OR correlat* OR condition* OR differ* OR relation* OR likely OR risk* OR associat* OR symptom* OR influen*)) OR sociodemograph* OR socio-demograph* OR lifestyle OR life-style OR financial* OR mobilit* OR impairment* OR balance OR adl OR auditor* OR visual* OR grip OR strength OR fatigue OR memory OR depress* OR anxi* OR energy-reserve OR vulnerab* OR perform* OR stress OR coping OR lonel* OR Resilien* OR income* OR socioeconomic* OR education* OR determinant* OR cognit* OR (postur* NEAR/3 instab*) OR equilibr* OR (characterist* NEAR/3 discriminat*) OR (daily NEAR/3 (life OR living) NEAR/6 activit*) OR ((physical* OR motor*) NEAR/3 activ*) OR disab* OR walking OR risk OR risks OR (self NEXT/1 (rat* OR report*)) OR subjectiv* OR objectiv* OR questionnaire* OR comorbid* OR scale OR score OR assessment* OR (social NEAR/3 (status* OR class* OR background* OR behav* OR identi*)) OR poverty OR pension* OR ((male* OR men) NEAR/3 (female* OR women) NEAR/6 (differen* OR more OR less OR likel*))):ab,ti)

Medline Ovid 2095

("Independent Living"/ OR "Residence Characteristics"/ OR (((independent* OR at-home) ADJ3 living) OR (aging ADJ3 place) OR (communit* ADJ6 (dwell* OR living OR resid*)) OR "liv* alone").ab,ti.) AND ("Frail Elderly"/ OR (frail*).ab,ti.) AND ("Biological Factors"/ OR "Social Class"/ OR exp "Social Behavior"/ OR "psychology".xs. OR demography/ OR Age Factors/ OR sex/ OR "Sex Characteristics"/ OR "Social Determinants of Health"/ OR "Income"/ OR exp "Sociological Factors"/ OR "Posture"/ OR exp depression/ OR exp "anxiety"/ OR exp "Cognition Disorders"/ OR exp "cognition"/ OR "Activities of Daily Living"/ OR "Motor Activity"/ OR Disability Evaluation/ OR "Disabled Persons"/ OR "Mobility Limitation"/ OR "walking"/ OR "comorbidity"/ OR exp risk/ OR "self concept"/ OR "self report"/ OR exp questionnaires/ OR (((biological* OR physical* OR social* OR psycholog* OR functional* OR clinical* OR living OR residence* OR age OR gender OR sex OR psychiat* OR neuropsychiat* OR sociological*) ADJ10 (factor* OR aspect* OR characteristic* OR correlat* OR condition* OR differ* OR relation* OR likely OR risk* OR associat* OR symptom* OR influen*)) OR sociodemograph* OR socio-demograph* OR lifestyle OR life-style OR financial* OR mobilit* OR impairment* OR balance OR adl OR auditor* OR visual* OR grip OR strength OR fatigue OR memory OR depress* OR anxi* OR energy-reserve OR vulnerab* OR perform* OR stress OR coping OR lonel* OR Resilien* OR income* OR socioeconomic* OR education* OR determinant* OR cognit* OR (postur* ADJ3 instab*) OR equilibr* OR (characterist* ADJ3 discriminat*) OR (daily ADJ3 (life OR living) ADJ6 activit*) OR ((physical* OR motor*) ADJ3 activ*) OR disab* OR walking OR risk OR risks OR (self ADJ (rat* OR report*)) OR subjectiv* OR objectiv* OR questionnaire* OR comorbid* OR scale OR score OR assessment* OR (social ADJ3 (status* OR class* OR background* OR behav* OR identi*)) OR poverty OR pension* OR ((male* OR men) ADJ3 (female* OR women) ADJ6 (differen* OR more OR less OR likel*))).ab,ti.)

PsycINFO Ovid 496

((((independent* OR at-home) ADJ3 living) OR (aging ADJ3 place) OR (communit* ADJ6 (dwell* OR living OR resid*)) OR "liv* alone").ab,ti.) AND ((frail*).ab,ti.) AND (exp "psychology"/ OR exp "depression (emotion)"/ OR exp "anxiety"/ OR exp "anxiety disorders"/ OR exp "Cognitive Impairment"/ OR exp "cognition"/ OR "self-concept"/ OR (((biological* OR physical* OR social* OR psycholog* OR functional* OR clinical* OR living OR residence* OR age OR gender OR sex OR psychiat* OR neuropsychiat* OR sociological*) ADJ10 (factor* OR aspect* OR characteristic* OR correlat* OR condition* OR differ* OR relation* OR likely OR risk* OR associat* OR symptom* OR influen*)) OR sociodemograph* OR socio-demograph* OR lifestyle OR life-style OR financial* OR mobilit* OR impairment* OR balance OR adl OR auditor* OR visual* OR grip OR strength OR fatigue OR memory OR depress* OR anxi* OR energy-reserve OR vulnerab* OR perform* OR stress OR coping OR lonel* OR Resilien* OR income* OR socioeconomic* OR education* OR determinant* OR cognit* OR (postur* ADJ3 instab*) OR equilibr* OR (characterist* ADJ3 discriminat*) OR (daily ADJ3 (life OR living) ADJ6 activit*) OR ((physical* OR motor*) ADJ3 activ*) OR disab* OR walking OR risk OR risks OR (self ADJ (rat* OR report*)) OR subjectiv* OR objectiv* OR questionnaire* OR comorbid* OR scale OR score OR assessment* OR (social ADJ3 (status* OR class* OR background* OR behav* OR identi*)) OR poverty OR pension* OR ((male* OR men) ADJ3 (female* OR women) ADJ6 (differen* OR more OR less OR likel*))).ab,ti.)

CINAHL EBSCOhost 901

(MH "Community Living+" OR AB (((independent* OR at-home) N2 living) OR (aging N2 place) OR (communit* N5 (dwell* OR living OR resid*)) OR "liv* alone") OR TI (((independent* OR at-home) N2 living) OR (aging N2 place) OR (communit* N5 (dwell* OR living OR resid*)) OR "liv* alone")) AND ("Frail Elderly+" OR MH "Frailty Syndrome" OR TI (frail*) OR AB (frail*)) AND (MH "Biological Factors+" OR MH "Social Class+" OR MH "Social Behavior+" OR MH demography OR MH Age Factors OR MH sex OR MH "Social Determinants of Health" OR MH "Income" OR MH "Posture" OR MH depression+ OR MH "anxiety+" OR MH "Cognition Disorders+" OR MH "cognition+" OR MH "Activities of Daily Living+" OR MH "Motor Activity+" OR MH Disability Evaluation+ OR MH "Physical Mobility+" OR MH "walking+" OR MH "comorbidity" OR MH "risk factors+" OR "self concept+" OR "self report+" OR MH questionnaires+ OR TI (((biological* OR physical* OR social* OR psycholog* OR functional* OR clinical* OR living OR residence* OR age OR gender OR sex OR psychiat* OR neuropsychiat* OR sociological*) N9 (factor* OR aspect* OR characteristic* OR correlat* OR condition* OR differ* OR relation* OR likely OR risk* OR associat* OR symptom* OR influen*)) OR sociodemograph* OR socio-demograph* OR lifestyle OR life-style OR financial* OR mobilit* OR impairment* OR balance OR adl OR auditor* OR visual* OR grip OR strength OR fatigue OR memory OR depress* OR anxi* OR energy-reserve OR vulnerab* OR perform* OR stress OR coping OR lonel* OR Resilien* OR income* OR socioeconomic* OR education* OR determinant* OR cognit* OR (postur* N2 instab*) OR equilibr* OR (characterist* N2 discriminat*) OR (daily N2 (life OR living) N5 activit*) OR ((physical* OR motor*) N2 activ*) OR disab* OR walking OR risk OR risks OR (self N1 (rat* OR report*)) OR subjectiv* OR objectiv* OR questionnaire* OR comorbid* OR scale OR score OR assessment* OR (social N2 (status* OR class* OR background* OR behav* OR identi*)) OR poverty OR pension* OR ((male* OR men) N2 (female* OR women) N5 (differen* OR more OR less OR likel*))) OR AB (((biological* OR physical* OR social* OR psycholog* OR functional* OR clinical* OR living OR residence* OR age OR gender OR sex OR psychiat* OR neuropsychiat* OR sociological*) N9 (factor* OR aspect* OR characteristic* OR correlat* OR condition* OR differ* OR relation* OR likely OR risk* OR associat* OR symptom* OR influen*)) OR sociodemograph* OR socio-demograph* OR lifestyle OR life-style OR financial* OR mobilit* OR impairment* OR balance OR adl OR auditor* OR visual* OR grip OR strength OR fatigue OR memory OR depress* OR anxi* OR energy-reserve OR vulnerab* OR perform* OR stress OR coping OR lonel* OR Resilien* OR income* OR socioeconomic* OR education* OR determinant* OR cognit* OR (postur* N2 instab*) OR equilibr* OR (characterist* N2 discriminat*) OR (daily N2 (life OR living) N5 activit*) OR ((physical* OR motor*) N2 activ*) OR disab* OR walking OR risk OR risks OR (self N1 (rat* OR report*)) OR subjectiv* OR objectiv* OR questionnaire* OR comorbid* OR scale OR score OR assessment* OR (social N2 (status* OR class* OR background* OR behav* OR identi*)) OR poverty OR pension* OR ((male* OR men) N2 (female* OR women) N5 (differen* OR more OR less OR likel*))))

Cochrane 230

((((independent* OR at-home) NEAR/3 living) OR (aging NEAR/3 place) OR (communit* NEAR/6 (dwell* OR living OR resid*)) OR 'liv* alone'):ab,ti) AND ((frail*):ab,ti) AND ((((biological* OR physical* OR social* OR psycholog* OR functional* OR clinical* OR living OR residence* OR age OR gender OR sex OR psychiat* OR neuropsychiat* OR sociological*) NEAR/10 (factor* OR aspect* OR characteristic* OR

correlat* OR condition* OR differ* OR relation* OR likely OR risk* OR associat* OR symptom* OR influen*)) OR sociodemograph* OR socio-demograph* OR lifestyle OR life-style OR financial* OR mobilit* OR impairment* OR balance OR adl OR auditor* OR visual* OR grip OR strength OR fatigue OR memory OR depress* OR anxi* OR energy-reserve OR vulnerab* OR perform* OR stress OR coping OR lonel* OR Resilien* OR income* OR socioeconomic* OR education* OR determinant* OR cognit* OR (postur* NEAR/3 instab*) OR equilibr* OR (characterist* NEAR/3 discriminat*) OR (daily NEAR/3 (life OR living) NEAR/6 activit*) OR ((physical* OR motor*) NEAR/3 activ*) OR disab* OR walking OR risk OR risks OR (self NEXT/1 (rat* OR report*)) OR subjectiv* OR objectiv* OR questionnaire* OR comorbid* OR scale OR score OR assessment* OR (social NEAR/3 (status* OR class* OR background* OR behav* OR identi*)) OR poverty OR pension* OR ((male* OR men) NEAR/3 (female* OR women) NEAR/6 (differen* OR more OR less OR likel*))):ab,ti)

Web of science 1787

TS=(((((independent* OR at-home) NEAR/2 living) OR (aging NEAR/2 place) OR (communit* NEAR/5 (dwell* OR living OR resid*)) OR "liv* alone")) AND ((frail*)) AND ((((biological* OR physical* OR social* OR psycholog* OR functional* OR clinical* OR living OR residence* OR age OR gender OR sex OR psychiat* OR neuropsychiat* OR sociological*) NEAR/9 (factor* OR aspect* OR characteristic* OR correlat* OR condition* OR differ* OR relation* OR likely OR risk* OR associat* OR symptom* OR influen*)) OR sociodemograph* OR socio-demograph* OR lifestyle OR life-style OR financial* OR mobilit* OR impairment* OR balance OR adl OR auditor* OR visual* OR grip OR strength OR fatigue OR memory OR depress* OR anxi* OR energy-reserve OR vulnerab* OR perform* OR stress OR coping OR lonel* OR Resilien* OR income* OR socioeconomic* OR education* OR determinant* OR cognit* OR (postur* NEAR/2 instab*) OR equilibr* OR (characterist* NEAR/2 discriminat*) OR (daily NEAR/2 (life OR living) NEAR/5 activit*) OR ((physical* OR motor*) NEAR/2 activ*) OR disab* OR walking OR risk OR risks OR (self NEAR/1 (rat* OR report*)) OR subjectiv* OR objectiv* OR questionnaire* OR comorbid* OR scale OR score OR assessment* OR (social NEAR/2 (status* OR class* OR background* OR behav* OR identi*)) OR poverty OR pension* OR ((male* OR men) NEAR/2 (female* OR women) NEAR/5 (differen* OR more OR less OR likel*)))))

Google scholar 200

"independent living"|"aging in place"|"community dwelling|dwellers" frail|frailty "biological|physical|social|psychological factors|aspects|characteristics"|sociodemographic|socioeconomics|determinants|"social status|class|background"|poverty|income
